# Biomechanical Evaluation of the Sheep Common Peroneal Nerve After Crush Injury

**DOI:** 10.3390/ani15050627

**Published:** 2025-02-21

**Authors:** Rui Alvites, Bruna Lopes, Ana Catarina Sousa, Fábio Pinheiro, Elisabete Silva, Justina Prada, Artur Varejão, Ana Colette Maurício

**Affiliations:** 1 Centro de Estudos de Ciência Animal (CECA), Instituto de Ciências, Tecnologias e Agroambiente da Universidade do Porto (ICETA), Rua D. Manuel II, Apartado 55142, 4051-401 Porto, Portugal; ruialvites@hotmail.com (R.A.); bilopes@icbas.up.pt (B.L.); anacatarinasoaressousa@hotmail.com (A.C.S.); 2 Departamento de Clínicas Veterinárias, Instituto de Ciências Biomédicas de Abel Salazar (ICBAS), Universidade do Porto (UP), Rua de Jorge Viterbo Ferreira, n° 228, 4050-313 Porto, Portugal; fpinheiro@inegi.up.pt; 3 Associate Laboratory for Animal and Veterinary Science (AL4AnimalS), 1300-477 Lisboa, Portugal; jprada@utad.pt (J.P.); avarejao@utad.pt (A.V.); 4 Instituto Universitário de Ciências da Saúde (IUCS), Cooperativa de Ensino Superior Politécnico e Universitário (CESPU), Avenida Central de Gandra 1317, 4585-116 Gandra PRD, Portugal; 5 Associated Laboratory for Energy, Transports and Aerospace (LAETA), Institute of Science and Innovation in Mechanical and Industrial Engineering (INEGI), 4200-465 Porto, Portugal; mesilva@inegi.up.pt; 6 Centro de Ciência Animal e Veterinária (CECAV), Universidade de Trás-os-Montes e Alto Douro (UTAD), Quinta de Prados, 5001-801 Vila Real, Portugal; 7 Departamento de Ciências Veterinárias, Universidade de Trás-os-Montes e Alto Douro (UTAD), Quinta de Prados, 5001-801 Vila Real, Portugal; 8 Neurology Service, Veterinary Hospital of the University of Trás-os-Montes e Alto Douro (UTAD), Quinta de Prados, 5000-801 Vila Real, Portugal

**Keywords:** axonotmesis, biomechanical behavior, common peroneal nerve, peripheral nerve, sheep, regenerative medicine

## Abstract

Axonotmesis, a common peripheral nerve injury in humans and animals, leads to significant biomechanical and physiological consequences. The lack of a standardized crushing protocol for complex animal models limits research and therapeutic translations for humans and clinically relevant animal species. This study aimed to assess the impact of different crushing forces on the biomechanical behavior of the sheep common peroneal nerve and to establish a force for standardized in vivo protocols. Fourteen nerves of equal length were harvested and preserved and their initial diameter measured. They were subjected to crushing forces of 0 N, 80 N, and 180 N for one minute. Post crushing, the diameter, ultimate tensile strength, displacement at rupture, stress, strain, and stiffness were evaluated. Results showed that increasing crushing forces significantly affected nerve biomechanical parameters. Nerves crushed with 180N displayed lower tensile strength, displacement, and stiffness but higher stress and strain, indicating greater physical damage and structural degradation. These findings suggest that 180N induces substantial nerve fiber rupture and disruption of nerve trunk support elements, making it a candidate force for an axonotmesis protocol in the ovine model. Future in vivo studies should validate its effectiveness in creating complete crush injuries with functional and histological consequences, facilitating protocol standardization and translational research.

## Simple Summary

Axonotmesis, a type of nerve damage caused by crushing, often leads to severe consequences in humans and animals. However, the lack of a standardized method for creating crush injuries in animal models makes it difficult for researchers to compare results and develop effective treatments for both humans and animals. This study explored how different levels of force affect the behavior of sheep nerves, aiming to establish a reliable protocol for future research. Nerves were collected, measured, and subjected to various crushing forces for one minute. Afterward, researchers measured their strength, flexibility, and structural changes. The results showed that higher forces caused more significant damage, reducing the strength and flexibility of the nerves while increasing the stress they could endure before breaking. The study identified 180 N as the most effective force to create consistent nerve damage, making it suitable for use in live animal studies. This research will help standardize methods for studying nerve injuries, improving the ability to test new treatments and potentially benefiting people and animals suffering from nerve damage. Standardized methods like this are essential for advancing medical and veterinary treatments applied to peripheral nerve regeneration.

## 1. Introduction

Peripheral nerve injury (PNI) is a common occurrence resulting in functional, physiological, and psychological consequences that are the target of frequent clinical approaches in both human medicine and veterinary medicine. It is estimated that cases of PNI occur in around 3% of trauma patients [1] and that around 13 to 23 per 1,00,000 people suffer from this condition [2]. The causes behind this type of injury are multiple and can range from external trauma, penetrating injuries, neoplasms, and metabolic changes to iatrogenic interventions such as surgery or perineural administrations [3]. The deeper nerves protected by surrounding structures tend to be affected by more aggressive injuries in cases of bone fractures, perforating damage, or the development of compressive masses; the more superficial nerves, such as the radial or common peroneal nerve, in part of their course, can easily be externally compressed and suffer a crushing injury. In particularly severe cases, when the nerve injury is just an additional element in a multiple trauma scenario, PNI is often undervalued or addressed late, missing the small window of opportunity that guarantees the success of a therapeutic approach, be it surgical or medical [4]. This reality gains even more importance in veterinary medicine, where diagnostic tools available in a timely manner and at affordable prices are even more scarce.

Therapeutic options for treating PNI cases range from conservative treatment to drug approaches and surgical interventions. The choice of the method to use will depend on several factors such as the type of injury, the injured nerve, the lesion location within the nerve, the state of the surrounding tissues, the time elapsed since the injury and, last but not least, the options available [5]. In complete transection injuries, a more aggressive type of injury with no capacity for spontaneous recovery, where there is a total loss of nerve continuity with affection not only of the axons but also of all the nerve envelopes of myelin and connective tissue [6], surgical techniques such as end-to-end sutures and neurografts continue to be the gold-standard approaches [7]. In crush injuries, since nerve continuity is maintained with different levels of loss of connective tissue coverings, spontaneous recovery is possible, although slow [6,8]. In these cases, conservative treatments and neurorehabilitation takes on greater importance [9]. Whatever the type of injury, no method can currently be considered as ideal, and the results tend to be imperfect, with long-term motor, sensory, and autonomic impairment and loss of quality of life for the patient.

Regenerative medicine is an alternative medical field that aims to establish innovative therapeutic approaches to medical problems whose surgical, medicinal, or conservative resolution is currently unsatisfactory [10]. The promotion of peripheral nerve regeneration has been the focus of multiple studies over the last decade [11]. The therapeutic fields explored within the PNI field are multiple, ranging from the development of new biomaterials that function as scaffolds to replace grafts and neural tube guides as physical bridges to guide and protect the site of regeneration to cell-based therapies that promote regeneration of the peripheral nerve while modulating the local and systemic immune response. More specifically, combined therapies, which use two or more pro-regenerative methods simultaneously, have gained special attention. The need to test therapies developed in animal models before applying them in real clinical scenarios has led to the use of traditional animal models such as rats, mice and rabbits, on which the overwhelming majority of work published in the last decade is based [12]. In these models, the lesional paradigms explored are essentially neurotmesis and axonotmesis, corresponding to complete transection and crush injuries, with the animals being evaluated regarding their functional recovery in vivo and the structural reorganization of the intervened nerves and effector muscles postmortem [8]. Advances in the area have been notable, but there is an excess of work developed in low-complexity animal models with little capacity for direct translation into clinical reality. Even though more complex animal models such as dogs or pigs are considered, they face ethical and emotional constraints that limit their wider use [12]. More recently, sheep have gained attention as an advantageous model for studying regeneration after PNI [13,14] due to availability, low price, ease of maintenance and handling, affable behavior, and fewer ethical restrictions. Furthermore, the position, distribution, functions, and dimensions of peripheral nerves as well as the functional consequences of their injury are identical to those observed in humans. The larger nerve diameter in sheep allows for a more precise and reproducible evaluation of injury and regeneration, closely mirroring human peripheral nerve dimensions. Additionally, the polyfasciculated structure of the sheep common peroneal nerve more accurately reflects the complexity of human nerves compared to the simpler nerve architecture observed in rodents. The greater similarity in fascicular organization and fiber distribution enhances the predictability of regenerative outcomes, reducing the variability seen in smaller models. Furthermore, the biomechanical properties of ovine peripheral nerves, including tensile strength and elasticity, more closely resemble those of humans, making them an ideal candidate for studying nerve repair techniques and biomaterial integration. These attributes enhance the ability to study axonal regeneration, neurophysiological responses, and the efficacy of therapeutic interventions in a setting that is more representative of human clinical scenarios [13].

Our research group has been developing new treatments for PNI over the last two decades, mainly exploring combined therapies based on the use of biomaterials and mesenchymal stem cells. Most of the advances achieved were made in vitro and in the rat model [15,16], but recently, the ovine model began to be explored, focusing on the common peroneal nerve instead of the sciatic nerve traditionally used in rodents. This polyfasciculated nerve is one of the branches of the sciatic nerve, with the advantage of having a superficial position in part of its route along the lateral side of the hindlimb, which facilitates surgical access for inducing injuries and for therapeutic approaches. The functional consequences of its injury are not as serious and deleterious to the animal’s quality of life as a sciatic nerve injury, allowing the animal to maintain the ability to bear weight and explore the environment, while its mixed nature leads to motor and sensory changes that can be easily observed and quantified over time [13,17]. In a previous work, a protocol for the ovine model was established and validated, including the establishment of a method for surgical access and induction of nerve lesions of neurotmesis and axonotmesis in the common peroneal nerve, for the application of therapeutic approaches, for methods of functional evaluation, and for ideal times for monitoring animals after injury. Subsequently, the intervened nerves were also collected and stereologically evaluated, with preliminary evaluation of the recovery behavior of the injured nerves after subjection to the established treatments. This assay allowed to obtain control values for both functional tests and stereological studies in animals subject to neurotmesis, which can now be used for comparison in future tests. Despite efforts, in animals subject to axonotmesis injuries, a complete descriptive study was not carried out since stereologically, after inducing crushing with a clamp exerting a pressure of 80 N, the observation of some degenerated/regenerated fascicles in conjunction with healthy ones indicated that the induced crush injury was not effective from the structural point of view, even if it was translated into compatible symptoms in vivo [13].

These results left open the standardization of an axonotmesis injury protocol in the ovine model, in which it is necessary to establish a new value of exerted pressure, greater than 80 N, to guarantee the complete crushing of the nerve fibers of the common peroneal nerve. From a 3Rs perspective, and to avoid using new animals for this purpose, it was decided to carry out a preliminary biomechanical characterization study of the sheep’s common peroneal nerve before moving on to new in vivo studies. The aim of this work was to study and compare the biomechanical behavior of the sheep common peroneal nerve after crushing by different pressures to establish which parameters will be used in the future in in vivo assays to induce effective axonotmesis injuries. Unlike previous studies that primarily relied on rodent models, this research intends to expand the understanding of peripheral nerve injury by incorporating a large animal model with greater anatomical and physiological similarities to humans. By investigating the biomechanical properties of the ovine common peroneal nerve under controlled crush conditions, this study may provide a more accurate representation of the forces required to induce standardized axonotmesis injuries and bridge the gap between small-animal research and human clinical applications, setting the stage for more effective translational studies.

## 2. Materials and Methods

### 2.1. Animals

The procedures using animals were previously approved by the Organism Responsible for Animal Welfare (ORBEA) of the Abel Salazar Institute for Biomedical Sciences (ICBAS) from the University of Porto (UP) (project 459/2023/ORBEA) and by the Veterinary Authorities of Portugal (DGAV) (project DGAV: 2018-07-11014510). All activities described took place in facilities previously approved by the official Portuguese authorities (Clinical and Veterinary Research Center of Vairão-CCIVV). The euthanasia and sample collection processes followed the principles present in the Portuguese decree law DL 113/2013, adapted directly from the EU directive 2010/63/EU of the European Parliament, and the OECD Guidance Document on the Recognition, Assessment and Use of Clinical Signs as Humane Endpoints for Experimental Animals Used in Safety Evaluation (2000). Furthermore, all measures were taken to avoid and minimize discomfort or pain in accordance with the humane endpoints for animal suffering and distress. The animals used to collect samples were not euthanized specifically for this work, and following the principles of Replacement and Reduction concerning individuals in animal experimentation, the samples were collected from animals euthanized in the context of other scientific studies in which there was no relationship or interference with the characteristics of the peripheral nerve, thus ensuring compliance with the assumptions of thoughtful use of animals for scientific purposes.

Samples were collected from fourteen sheep (*Ovis aries*) comprising females 5 to 6 years old, of the merino breed, and weighing 50–60 kg. The animals were purchased from national producers duly authorized and approved by the institution, ensuring the classification of the farm as brucellosis-free and the testing for infectious diseases prior to transport. After receiving the animals, we performed a general clinical examination and a prophylactic protocol for internal deworming, vaccination against enterotoxemia, corrective hoof trimming, and wool shearing. The animals were housed in social groups to allow the gregarious behavior of the species, and permanent access to water and feed with concentrate and hay was guaranteed in doses and frequencies adapted to established nutritional needs. Regularly throughout the stabling period and before euthanasia, the animals were subject to clinical evaluations to ensure their well-being.

#### Euthanasia and Sample Collection

**Figure 1:**
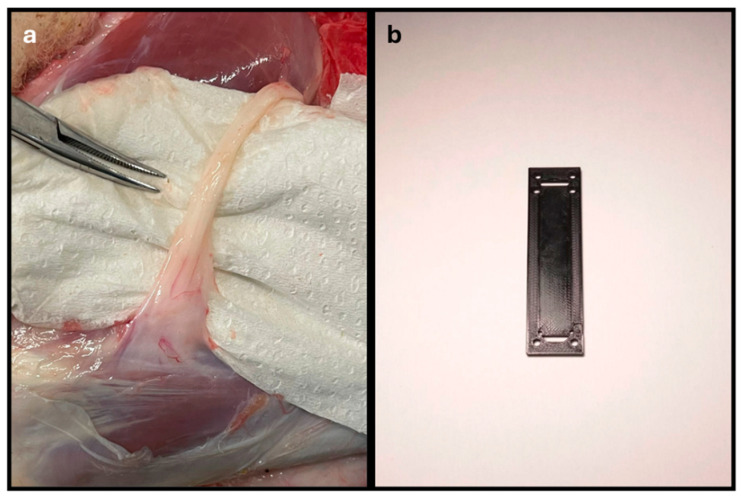
Process for collecting the common peroneal nerve in sheep: (**a**) common peroneal nerve duly individualized from neighboring tissues; (**b**) device used to collect the common peroneal nerve and to avoid curling, guaranteeing equal length for all samples (7 cm).

After the periods established in the respective scientific works, the animals were euthanized. The sheep were sedated with xylazine (Rampun^®^, Bayer, Leverkusen, Germany, 0.1 mg/Kg, IM) and butorphanol (Dolorex^®^, Merck Animal Health USA, Rahway, NJ, USA, 0.05 mg/Kg, IM) and then euthanized using an overdose of sodium pentobarbital (Eutasil^®^, Ceva Animal Health Solutions, Libourne, France, 100 mg/kg IV). After confirmation of death, the animals were placed in lateral decubitus on a surgical table, and a trichotomy of the proximal region of the hind limb was performed. Access to the common peroneal nerve was achieved as previously described [13]. Briefly, an incision was made at the level of the patella, which was extended along the tibia, in plantar position, ending 2 cm distally to the crest of the tibia. After the skin incision and subcutaneous debridement, the identification of the biceps femoris muscle allowed the observation of the common peroneal nerve arising underneath it. Next, the crural fascia was carefully debrided to allow individualization of the nerve from neighboring tissues (Figure 1a). An adapted device was used to immobilize the nerve, prevent curling, and allow the collection of a segment of equal dimensions (7 cm) for all samples (Figure 1b), and a small suture knot was applied proximally for subsequent nerve orientation. The nerves were preserved in 4% buffered formaldehyde for 48 h until further histological analysis. The nerves intended for biomechanical evaluation were subsequently transferred to phosphate-buffered saline (PBS) until the following tests.

### 2.2. Histological Evaluation

The common peroneal nerves were subjected to a routine histological processing after paraffin embedding. Sections of 4 mm thickness were stained with hematoxylin and eosin for structural histological evaluation. To identify connective tissue sheaths, sections were stained with Masson’s trichrome. To observe myelin sheaths, sections were stained with Lugol Fast Blue. All slides were evaluated by an experienced veterinary pathologist. A Nikon Eclipse E600 microscope with a Nikon DXM1200 digital camera (Nikon Instruments Inc., Melville, New York, NY, USA) was used for microscopic observations and image capture.

### 2.3. Nerve Crushing Test

**Figure 2:**
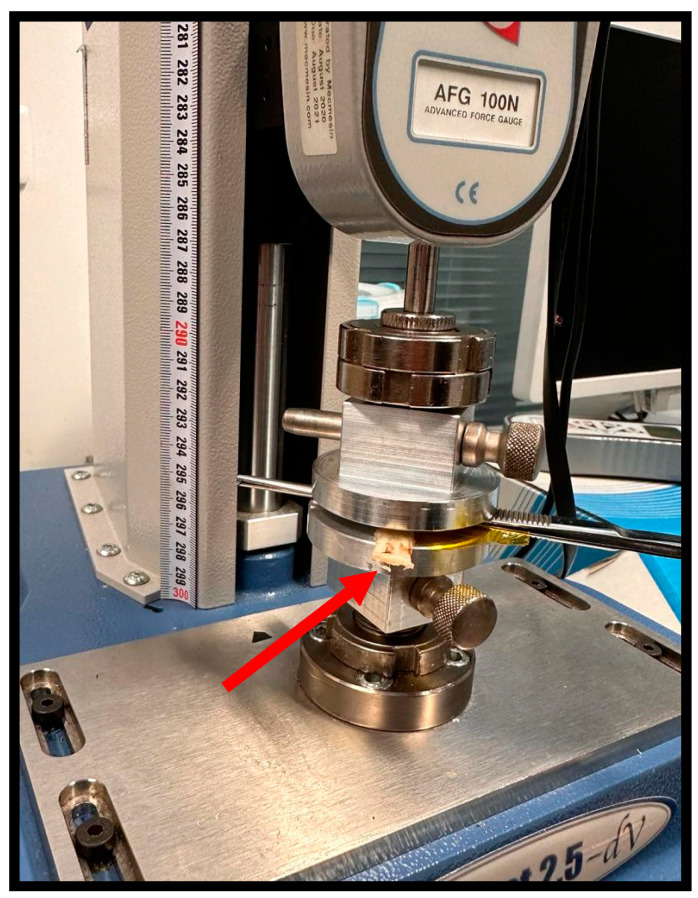
Process of crushing the sheep’s common peroneal nerve. The nerve was placed between the claws of the surgical forceps so that the application of a constant perpendicular force by the force tester platforms could induce crushing at the midpoint of the collected nerve segment to an extent of 5 mm. Red arrow: end of the common peroneal nerve under crushing.

Before the crushing procedures, the diameter of all nerves was measured using a micrometer. Considering that the collected samples were all of the same length, the measurement was made exactly in the middle of the collected nerve segment. The crushing operations were carried out with a Multitest 2.5 DV Motorized Force Tester (Mecmesin^®^ Horsham, Horsham, UK). To simulate the crushing to be induced intrasurgically with the 5 mm wide, non-serrated crush clamp [13], a 5 mm wide, non-serrated surgical forceps was used. The nerve was placed over the lower claw of the forceps and the set placed over the lower platform of the force tester. Then, a downward perpendicular compressive force was applied in such a way that the upper platform of the force tester pushed the upper claw of the forceps against the nerve, inducing the crushing injury between the two claws of the forceps. The nerve and clamps were arranged in such a way that the crushing force induced by the moving tester platforms was applied precisely to the middle of the harvested nerve segment (Figure 2). Two different compressive forces were applied and tested: 80 N (five nerves) and 180 N (five nerves). Four nerves were not crushed (0 N); these were considered as controls. Crushing forces were maintained for 1 min, as intended for intrasurgical application. Immediately after the crush, the nerves were released, and the presence of the crush site was confirmed. The diameter of the midpoint of the nerve was measured again after crushing to quantify the variation in comparison with the pre-crushing dimensions.

### 2.4. Biomechanical Tests

**Figure 3:**
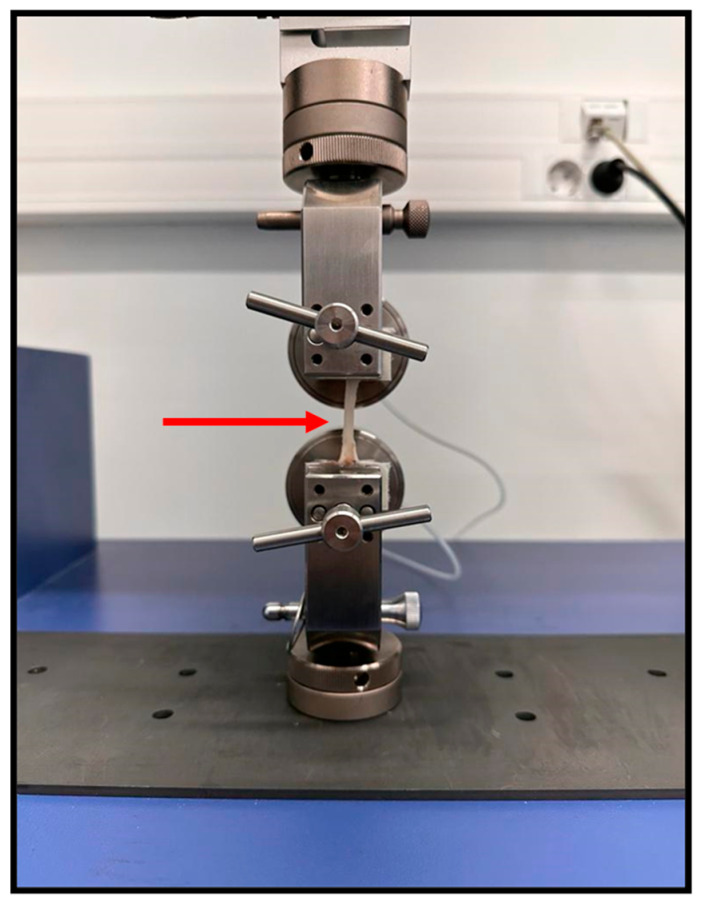
Biomechanical testing of the common peroneal nerve using the pulled-to-failure process. Both ends of the nerves were held between a pair of pneumatic grippers using a Velcro covering to prevent slippage. Red arrow: common peroneal nerve suspended between the pneumatic grippers, with a gouge length of 3.5 cm.

Between crushing and the remaining procedures, the nerves were kept in PBS to avoid dehydration. Biomechanical testing was performed using the Multitest 10-i Dual Column Strength Tester (Mecmesin^®^, Horsham, UK) and Emperor™ Force software (Version 1.18, Mecmesin^®^, Horsham, UK). Both ends of the nerves were held between a pair of pneumatic grippers using a Velcro covering to prevent slippage. An average gouge length of 3.5 cm was used (Figure 3). The common peroneal nerves subject to the different crushing forces (0 N, 80 N, and 180 N) were then pulled to failure at a strain rate of 0.16 mm/s. The tensile load and displacement were recorded. The procedure was filmed to record the mechanisms of failure of the common peroneal nerves. A typical load–displacement curve was generated for each nerve evaluated.

### 2.5. Biomechanical Parameters

To evaluate and characterize the biomechanical behaviors of the common peroneal nerves after crushing with the different forces applied, the following diverse parameters were considered.

#### 2.5.1. Stress

Stress (*σ*) was determined in each nerve considering the applied force (*F*) and the crushing area (*A*), calculated based on the diameter measured at the central point of the nerve segment and the width of the claw of the surgical forceps. Equation (1) was used.

(1) $$\sigma =\frac{F}{A}$$

#### 2.5.2. Strain

Strain (*ε*) was calculated in each nerve after the crush induction, considering the initial (before crushing—*Di*) and final (after crushing—*Df*) diameters and the consequent variation in diameter (*ΔD*). Equation (2) was used.

(2) $$\varepsilon =\frac{\Delta D}{Di}\times 100$$

#### 2.5.3. Ultimate Tensile Strength

The ultimate tensile strength or maximum load (*F*) considered was the one at which failure in the common peroneal nerve was observed during the pulled-to-failure test.

#### 2.5.4. Stiffness

Stiffness (*k*) was calculated considering the load at which failure in the common peroneal nerve was observed during the pulled-to-failure test and the maximum displacement observed at that moment. Equation (3) was used.

(3) $$k=\frac{F}{\Delta x}$$

#### 2.5.5. Statistical Analysis

Statistical analysis was performed using the GraphPad Prism software version 9.00 for Windows (GraphPad Software, La Jolla, CA, USA). Whenever appropriate, data and results were expressed as mean ± SEM. Comparisons between groups in the results of different tests were based on the application of a parametric test. A value of *p* < 0.05 was considered as statistically significant. Significance of the results is shown according to *p* values by the symbol *: * corresponds to 0.01 ≤ *p* < 0.05, ** to 0.001 ≤ *p* < 0.01, *** to 0.0001 ≤ *p* < 0.001, and **** to *p* < 0.0001.

## 3. Results

### 3.1. Histological Evaluation Results

**Figure 4:**
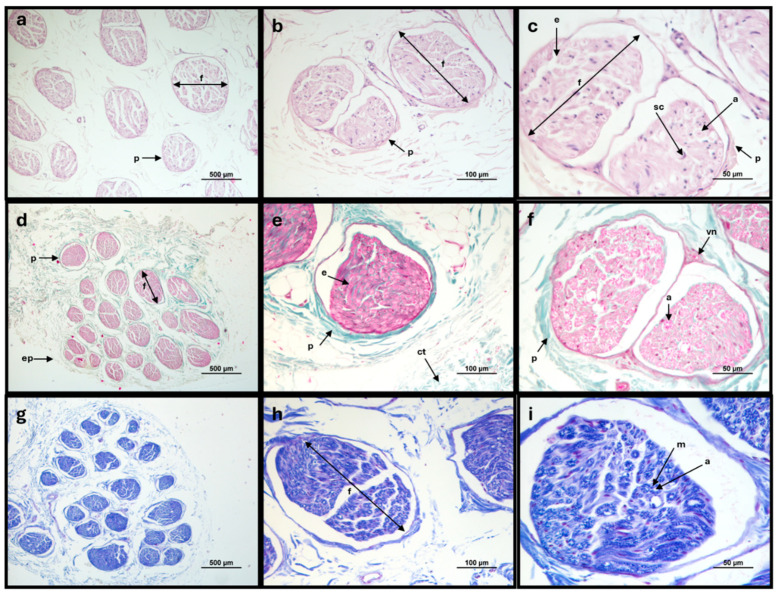
Histologic cross-section of the sheep common peroneal nerve: (**a**–**c**) Hematoxylin and Eosin stain; (**d**–**f**) Masson’s trichrome stain; (**g**–**i**) Lugol Fast Blue stain. Staining with Hematoxylin and Eosin allowed the identification of the ultrastructural characteristics of the peripheral nerve, with visualization of the nerve fascicles, connective tissue sheaths, nerve fibers, and Schwann cells around the myelinated fibers. Staining with Masson’s trichrome allowed to observe the epineurium, perineurium, and endoneurium in addition to abundant interfascicular connective tissue. Staining with Lugol Fast Blue allowed the observation of the myelin sheaths surrounding the nerve fibers. f, nerve fascicle; ep, epineurium; p, perineurium; e, endoneurium; ct, interfascicular connective tissue; a, axons; sc, Schwann cells; m, myelin sheath.

The different histological stains allowed the evaluation of the microscopic and ultrastructural morphological characteristics of the common peroneal nerves, confirming the polyfasciculated nature of this nerve and the histological normality of the collected samples. Staining with Hematoxylin and Eosin allowed visualization of the nerve fascicles involved by the perineurium, the individual axons involved by the myelin sheath and the presence of Schwan cells, responsible for myelination in the peripheral nerve. Masson’s trichrome staining allowed confirming the presence of connective tissue sheaths externally surrounding the entire nerve (epineurium), involving each nerve fascicle (perineurium) and each nerve fiber (endoneurium). The presence of abundant interfascicular connective tissue was also observed. Luxol Fast Blue, by staining the myelin sheaths, confirmed the abundant myelination of this nerve and the presence of myelin sheath around each nerve fiber. The vasa nervorum were also observed (Figure 4).

### 3.2. Nerve Crushing Test

**Figure 5:**
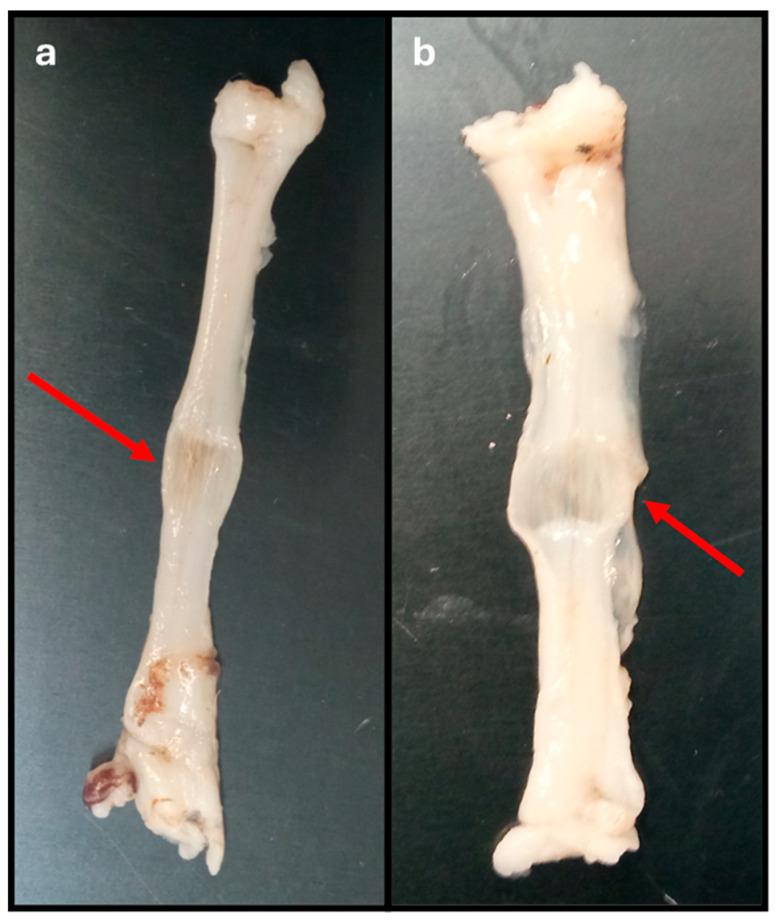
Common peroneal nerves after being subjected to crushing with different forces for one minute: (**a**) 80 N; (**b**) 180 N. Red arrow: crushing site, with flattening of the site subject to the crushing force and variation in the diameter of the nerve trunk.

The method used to induce crushing in the common peroneal nerves was effective and allowed the simulation of an axonotmesis injury in the nervous tissues used. The presence of a crushing site, with respective flattening of the nerve, and local variation in the diameter of the nerve trunk can be confirmed in Figure 5. In nerves subjected to a force of 180 N, a greater flattening and translucency of the crushing site and a greater increase in the final diameter were observed comparing to the diameter of the nerve trunk measured before crushing.

### 3.3. Biomechanical Tests

**Figure 6:**
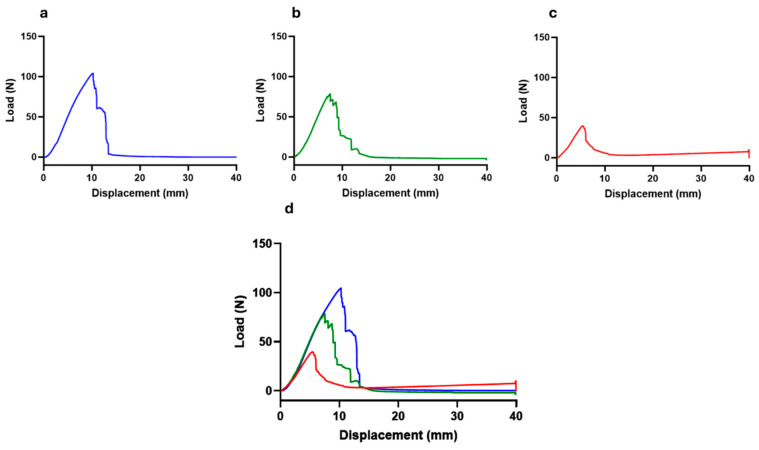
Load–displacement curves. (**a**) Nerves not subject to crushing (0 N—control); (**b**) nerves subject to a force of 80 N; (**c**) nerves subjected to a force of 180 N; (**d**) superposition of the three load–displacement curves.

The load–displacement curve obtained after biomechanical testing of nerves subjected to different crushing loads can be seen in Figure 6. The values of ultimate tensile strength (tensile strength at which the nerve rupture occurred) and displacement at rupture are found in Table 1.

The load–displacement curves are typical for all nerves, with the maximum peak of each graph representing the moment at which physical rupture of the common perineal nerve occurred. In each nerve, the graphic peak corresponds to the maximum force supported by the nerve during the pulled-to-failure movement (ultimate tensile strength), establishing the maximum distance of displacement recorded in the moment of the rupture. The ultimate tensile strength, that is, the maximum force supported by the nerves, was 109.59 ± 6.33 N for non-crushed nerves, 91.16 ± 9.19 N for nerves subjected to 80 N, and 49.38 ± 19.13 N for nerves subjected to 180 N of crushing. In the different crushing scenarios, this force corresponds to a displacement of 11.12 ± 1.30 mm, 8.78 ± 1.60 mm, and 6.26 ± 0.96 mm, respectively.

### 3.4. Biomechanical Parameters

#### 3.4.1. Stress

**Figure 7:**
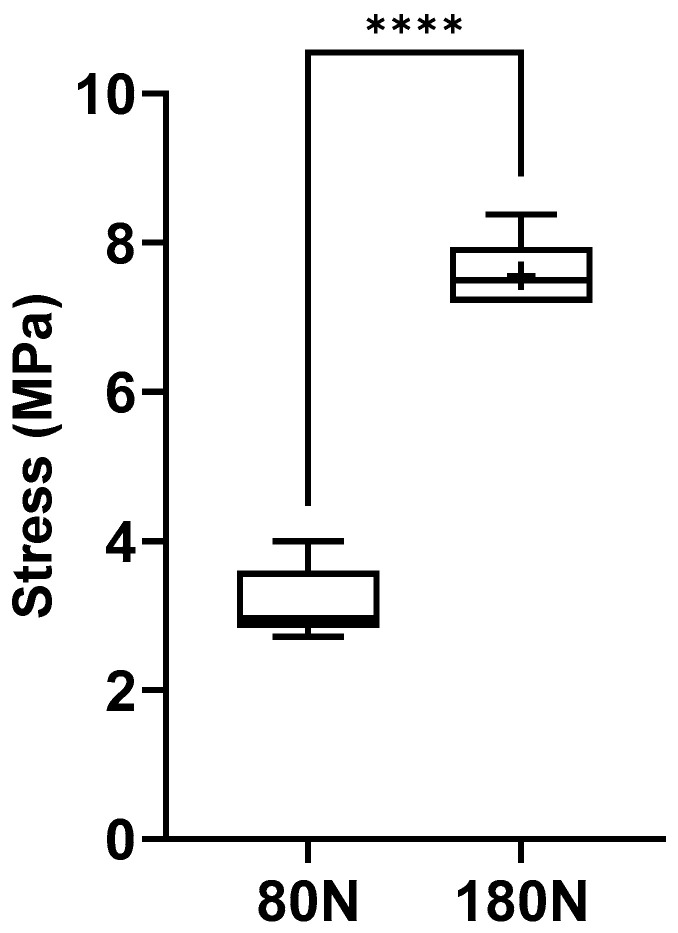
Stress of the common peroneal nerves during the crushing test. The plots represent the 25 percentile (Q1) and 75 percentile (Q3) values. The lower whiskers correspond to Q1 + 1.5 IQR and the upper whiskers Q3 + 1.5 IQR. +, mean value. * corresponds to 0.01 ≤ *p* < 0.05, ** to 0.001 ≤ *p* < 0.01, *** to 0.0001 ≤ *p* < 0.001, and **** to *p* < 0.0001.

The stress values, that is, the force applied per unit of area on each nerve, are graphically represented in Figure 7, with the corresponding values in Table 1. The stress was calculated for each nerve considering the measured diameter and the width of the claw of the surgical forceps used to induce crushing. The observed stress values were 3.17 ± 0.17 MPa for nerves subjected to a force of 80 N and 7.55 ± 0.43 MPa for those subjected to a force of 180 N. Statistical differences were observed between the groups (*p* < 0.0001). Nerves subjected to a higher crushing force were also those exposed to greater mechanical stress during crushing.

#### 3.4.2. Strain

**Figure 8:**
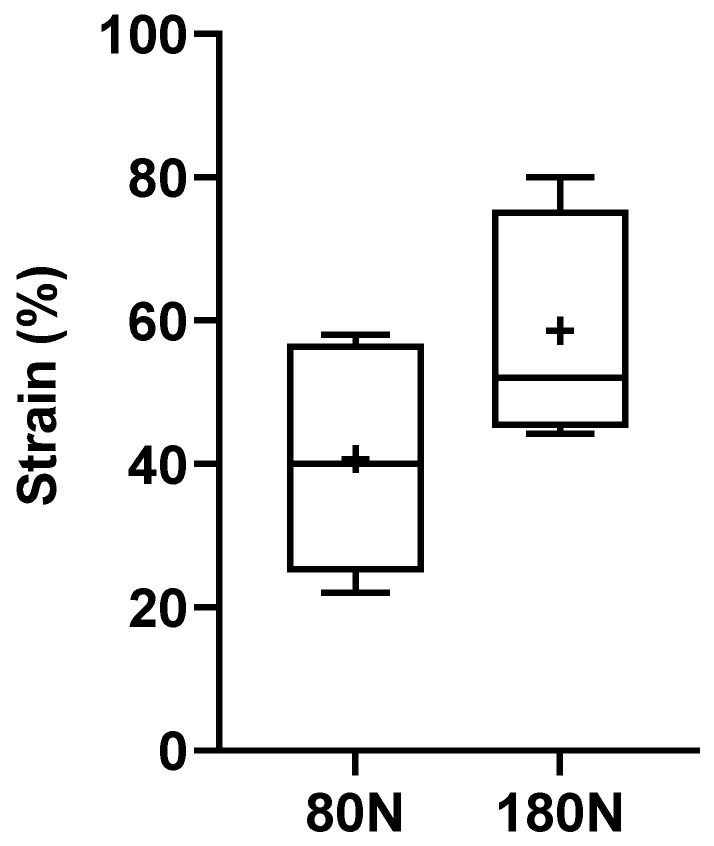
Strain of the common peroneal nerves during the crushing test. +, mean value. * corresponds to 0.01 ≤ *p* < 0.05, ** to 0.001 ≤ *p* < 0.01, *** to 0.0001 ≤ *p* < 0.001, and **** to *p* < 0.0001.

The % of deformation suffered by the nerves after the crushing force is graphically represented in Figure 8, with the corresponding values in Table 1. The strain was calculated considering the diameters calculated in the nerves before and after the application of the different crushing forces. The deformations observed were 40.67 ± 14.39% for nerves subjected to 80 N and 58.57 ± 14.30% for those subjected to 180 N. No statistical differences were observed between the two groups, although the global deformation observed was greater in nerves subjected to a higher crushing force, as seen in Figure 5.

#### 3.4.3. Ultimate Tensile Strength

**Figure 9:**
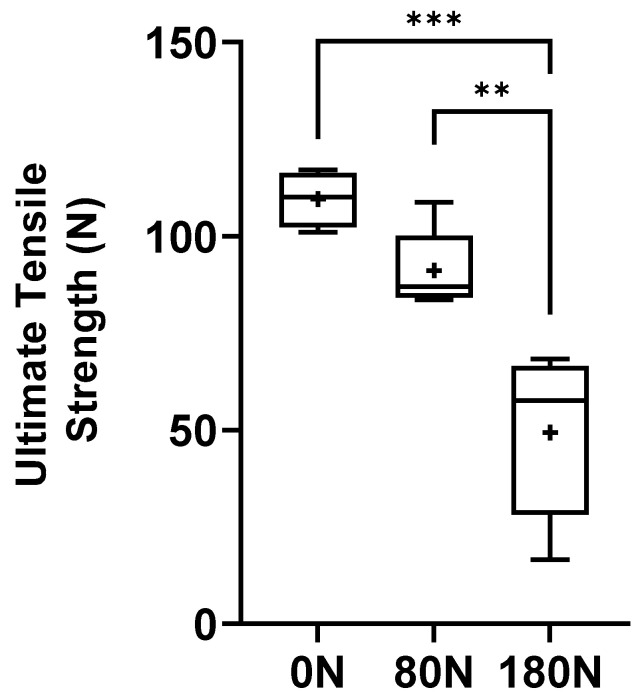
Ultimate tensile strength of the common peroneal nerves to rupture. +, mean value. * corresponds to 0.01 ≤ *p* < 0.05, ** to 0.001 ≤ *p* < 0.01, *** to 0.0001 ≤ *p* < 0.001, and **** to *p* < 0.0001.

The ultimate tensile strength values, that is, the tensile strength values at which the nerves subjected to different crushing forces suffered rupture, are graphically represented in Figure 9, with the corresponding values in Table 1. Statistical differences were observed between the nerves not crushed and between those subjected to 180 N (*p* = 0.0002) and those subjected to 80 N and 180 N (*p* = 0.0025). The non-crushed nerves were those that withstood a greater tensile force before rupturing, therefore suffering greater displacement during stretching. In contrast, the nerve subjected to a greater crushing force, 180 N, ruptured when subjected to a lower tensile force, resulting in lower displacement.

#### 3.4.4. Stiffness

**Figure 10:**
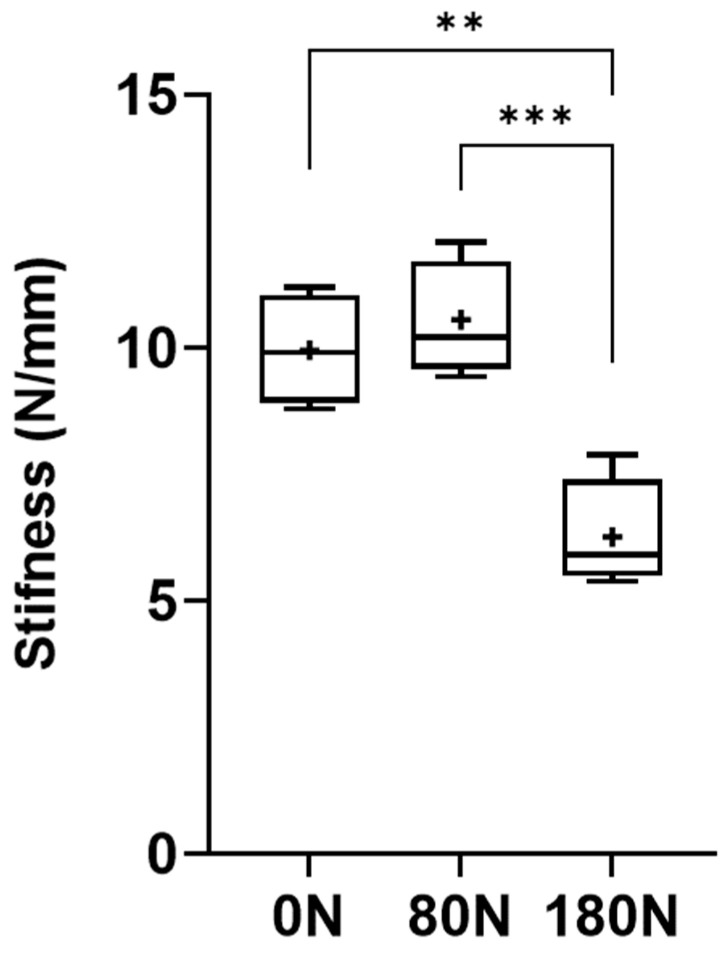
Stiffness of the common peroneal nerves under tensile testing. +, mean value. * corresponds to 0.01 ≤ *p* < 0.05, ** to 0.001 ≤ *p* < 0.01, *** to 0.0001 ≤ *p* < 0.001, and **** to *p* < 0.0001.

The stiffness values of the nerves are represented in Figure 10, with the corresponding values in Table 1. The stiffness was calculated considering the relationship between the ultimate tensile strength and the maximum displacement observed in the moment of rupture of the nerve trunk. The stiffness values observed were 9.95 ± 0.98 N/mm for intact nerves, 10.56 ± 1.00 N/mm for nerves subjected to 80N and 6.26 ± 0.96 N/mm for nerves subjected to 180 N. Statistical differences were observed between the nerves not crushed and those subjected to 180 N (*p* = 0.0023) and between those subjected to 80 N and 180 N (*p* = 0.0005). The lowest stiffness was observed in nerves subjected to a greater crushing force, with the greatest stiffness being recorded in those subjected to a crushing force of 80 N.

## 4. Discussion

The scientific literature has devoted much less attention to the study of peripheral nerve crush injuries than to complete transection ones. The crushing of a peripheral nerve is caused by an external compressive force or stretching, whether naturally occurring or iatrogenic, and this happens mainly in large-caliber nerves that are close to bone structures or that have a superficial course [8]. It has long been known that peripheral nerves undergo mechanical changes when subjected to crushing, which has important consequences on the integrity and functionality of the injured nerve [18]. In Seddon’s classification criteria, this type of injury is called axonotmesis and is characterized by a lesion that affects the axons and the myelin sheath, with the maintenance of the integrity in the connective tissue envelopes and support structures. In the Sunderland classification, axonotmesis is subdivided into three categories depending on whether the endoneurium and perineurium may also be involved, although the epineurium always remains intact [19]. The vasa nervorum can rupture with consequent ischemia, or tearing of the intraneural connective tissue can lead to hemorrhage and necrosis [20]. In any case, the occurrence of axonal injury leads to the development of the normal Wallerian degeneration sequence at the edges of the crushed area, with dragging of the ruptured nerve structures away from the point of greatest pressure and towards the limits of crushing [21], but the preservation of the nerve connective tissue allows regeneration to be guided with potential for full spontaneous recovery, even if it takes longer than a simpler neuropraxia injury. The functional consequences manifest distally to the site of injury with sensory, motor, and autonomic changes; pain and muscle wasting and symptoms compatible with retrograde degeneration may also be observed. Spontaneous nerve growth and regeneration generally occurs at a rate of 1 mm/day (approximately 30 mm per month), and regeneration and full recovery may take months or years to occur [22]. The degree of nerve damage in an axonotmesis injury is influenced by the applied pressure and the crushing duration [23]. Furthermore, the regenerative capacity in this type of injury depends on the extent of the lesion, the distance from the injury site to the cell body and to the effector organ, and the occurrence of additional complications in the surrounding tissues and organs. Generally, nerves with a simple branching pattern that are purely sensory or motor are more likely to regenerate successfully than mixed ones [24].

Due to its ability to regenerate spontaneously and the maintenance of nervous continuity, the therapeutic options developed for cases of axonotmesis are more limited than those available for neurotmesis injuries, often comprising conservative approaches such as physiotherapy or, in more complicated and severe cases, surgical approaches for nerve decompression [22,24]. Experimentally, options such as wrapping with biomaterials or cell-based therapies to protect and act directly on the crush site were also explored [25], but therapeutic options for these clinical situations are still scarce. Even considering that spontaneous regeneration occurs after a few months, the consequences and physical and psychological limitations of those affected during this period are relevant, and it is important to explore new therapeutic approaches.

As expected, the overwhelming majority of experimental studies on this topic are carried out in low-complexity animal models such as rats and mice [12], and the use of other models is rare [14,26]. In the past, Varejão et al. developed a method of inducing a sciatic nerve crushing injury in the rat model using a non-serrated clamp applying a force of 54 N for 30 s, resulting in an applied pressure of 9 MPa and in a 3 mm long lesion [27]. This model has been used frequently over the years, proving to be effective in this animal model. The standardization of axonotmesis models is not as well described in other species, a flaw that the authors consider essential to be addressed. From a translational perspective, and to allow the application of therapies already explored in the rat [15,16] in a more complex model before its application in clinical trials in humans or in veterinary species of clinical interest, our group developed neurotmesis and axonotmesis protocols to be applied in the common peroneal nerve of the ovine model [13]. For the axonotmesis model, adapting the protocol applied to the rat model, a non-serrated clamp was tested exerting a force of 80 N over 1 min, inducing a 5 mm lesion. Despite the manifestation of functional deficits after the injury, after 12 weeks of follow-up, this crushing did not translate into a histopathological scenario compatible with axonotmesis, with healthy fibers being identified in the middle of fibers undergoing regeneration. The crushing probably did not translate into an effective axonotmesis since, considering the force applied and the variation in the diameter of the nerve before and after the injury, the pressure exerted to the common peroneal nerve of the sheep was only 4 MPa. Additionally, the common peroneal nerve in this species is mixed and has a sizable diameter (which distributes the crushing force over a greater area), large amounts of connective tissue (providing additional structural resistance), and greater amounts of infiltrated adipose tissue than the nerves of the rat, which may contribute to nerve flexibility and resilience. With around 70% of the fibers in this nerve having less than 11 µm [13], integral crushing of all fibers is significantly more challenging. These factors align with previous findings in large animal models, where peripheral nerves exhibit greater stiffness and mechanical resistance compared to rodents [26]. For the same pressure to be exerted on the sheep’s common peroneal nerve, it would be necessary to apply a force of 180 N for the same period (1 min) and induce a lesion of equal extent (5 mm). Thus, the aim of this work was precisely to study ex vivo the biomechanical consequences observed in common peroneal nerves of the sheep when subjected to different crushing forces. In this way, it was possible to determine the force necessary to be applied in vivo without the need to use more animals before carrying out effective pre-clinical tests.

Previously, a study was performed using human digital nerves subjected to different crushing forces. In this work, a direct influence of the different crushing forces applied on the physical behavior of the nerves and on the biomechanical parameters considered was not identified [28]. An identical and adapted protocol was used here in the common peroneal nerve of the sheep.

The device developed for harvesting the common peroneal nerves, shown in Figure 1, proved to be effective in facilitating the harvesting of properly oriented nerves without curling and all with the same length of 7 cm. This facilitated the subsequent application of crushing forces exactly to the middle of the nerve, ensuring an equal distribution of forces at the midpoint and placing all nerves in equal physical conditions for evaluation.

The histological and immunohistochemical evaluation of the collected nerves allowed to observe the microscopic morphology expected for this peripheral nerve, with its polyfasciculated and myelinated nature. The amount of connective tissue is significant and appears to be proportionally higher when compared to other species, such as dogs [29]. A greater amount of connective tissue represents additional protection of the axons within the nerve trunk, which may help explain the difficulty in inducing an effective crush injury when using insufficient pressure. Additionally, macroscopically, a significant amount of adipose tissue infiltrated between the nerve envelopes is noticeable, which facilitates nerve sliding and makes it difficult to stabilize when using the non-serrated clamp. The presence of a significant amount of connective tissue and interfascicular adipose infiltration could contribute to the nerve’s increased mechanical resistance compared to smaller-caliber nerves, such as those in rodents. Finally, the diameter of the fibers identified in the ovine nerve appears to be smaller than the corresponding average diameter in the human nerve [30]. A greater proportion of small-diameter fibers with a greater amount of connective tissue and adipose tissue infiltration may justify the difficulty in inducing effective crushing of all fibers within the nerve trunk.

The crushing and pulled-to-failure tests proved to be effective in allowing assessments of the intended biomechanical parameters. The crushing test made it possible to effectively mimic the axonotmesis injury intended to be applied in vivo, with the application of two different forces, 80 and 180 N, for one minute and with an extension of 5 mm. Macroscopically, after crushing the nerve, it was possible to observe the translucent and enlarged crushed area, as observed in vivo [13]. In both applied forces, there was an increase in the diameter at the crushing site, which biomechanically translated into a variation in strain that represents a change in shape or size of a material under the action of a certain force. The deformation in the common peroneal nerves was higher in the nerves subjected to a force of 180 N, although it was without statistical differences compared to the group where the force of 80 N was applied. Statistically relevant were the differences observed in the stress to which the nerves were subjected during crushing, that is, the internal force that the material experiences when subjected to an external force. Stress was substantially higher in nerves subjected to a force of 180 N, with statistically significant differences compared to nerves subjected to 80 N.

The pulled-to-failure test aims to apply an increasing traction force over a material until rupture or irreversible deformation occurs. In this case, it allowed to determine at what level of traction force the nerve rupture occurred, considering that a crushed nerve with destruction of its internal support structures will have less resistance to rupture than an intact one. Nerves not subjected to crushing or subjected to a crushing force of 80 N ruptured later than those subjected to 180 N, with statistical differences being observed between the groups. Directly related to this parameter is stiffness, which determines the ability of a material to resist deformation, relating the ultimate tensile load force to the displacement observed at the moment of rupture. In this case, it was also in the nerves subjected to a force of 180 N that lower stiffness and resistance were observed, once again with statistical differences regarding the different conditions tested.

Unlike the studies developed by Wong et al. [28] on human digital nerves, in our study, the biomechanical tests and evaluations carried out demonstrate that compression has a clear and significant influence on the parameters evaluated, with an increase in strain and stress and a decrease in ultimate tensile strength and stiffness in the nerves subjected to a higher force, with statistical differences between groups. These differences may be explained in part by nerve size and fiber composition (the common peroneal nerve is polyfasciculated, while the digital nerves are monofasciculated); by connective tissue density, which influences resistance to compression; and by variability in crushing techniques, as force application methods differ across studies.

The absence of statistical differences in the different parameters between the control groups and the group subjected to 80 N, in addition to the histomorphological characteristics described above, justifies the ineffectiveness of axonotmesis when the animals were subjected to this crushing force in vivo. The ultimate tensile load value in the intact common peroneal nerve of the sheep is higher than the value observed for the same nerve in humans, where the stiffness was also inferior [31], reinforcing the greater mechanical resistance of this nerve in the sheep species, potentially due to a higher proportion of connective tissue and myelinated fibers. The value of 180 N, calculated so that a pressure of 9 MPa can be applied (the one that efficiently produces an axonotmesis lesion in the rat), effectively induces a change in all biomechanical parameters studied, and the statistical differences observed between the nerves subjected to this force and those crushed with 80 N appear to indicate that this force will be effective in creating a complete axonotmesis when applied to sheep nerves in vivo. As far as the authors know, there are no studies that have evaluated crushing forces of this magnitude in the sheep model. In the rat model, forces of 150 N allowed, after identification of post-crushing neurological deficits, full functional recovery over time [32]. Even considering the dimensional and biomechanical differences between species and the different forces applied, the same is expected to happen in sheep.

The biomechanical tests conducted in this study provided a precise assessment of the impact of different crushing forces on the ovine common peroneal nerve. The use of the Multitest 2.5 DV Motorized Force Tester for nerve crushing and the Multitest 10-i Dual Column Strength Tester for biomechanical evaluation ensures a high level of accuracy and repeatability in force application and mechanical property measurements. Additionally, the use of a 5 mm wide, non-serrated surgical forceps aimed to replicate the crushing induced in vivo, ensuring that the force was evenly distributed across the central section of the nerve. It is important, however, to list the limitations that can be considered. The most significant is the ex vivo nature of the tests, as the biomechanical properties of cadaveric nerves may differ from those of living tissues. Although samples were preserved in PBS to prevent dehydration, the absence of blood flow, cellular metabolism, and interaction with surrounding tissues could influence the mechanical response of the nerve. Consequently, values for resistance, deformation, and rupture may not perfectly reflect in vivo conditions. Another potential limitation is the variability in nerve diameter, which, despite random distribution among groups, could affect the uniformity of force distribution during compression and tensile testing. Additionally, while the force application method was designed to ensure consistency, slight variations in the positioning of the forceps or nerve fixation may have introduced minor deviations in stress distribution and deformation measurements. Despite these limitations, the statistical differences observed between the different test conditions suggest that the applied forces were sufficient to induce significant and measurable biomechanical changes.

The crushing force established here should in the future be tested under different conditions to confirm its effectiveness. In an initial phase, and once again following the assumptions of the 3Rs principles for more ethical use of animals in product testing and scientific research, the authors intend to use computational modeling that can be employed to predict in vivo mechanical effects, complementing experimental approaches. With these simulation models, it is possible to simulate the pressure distribution and model the interaction between the nerve and the surrounding tissues under different loading conditions, making it possible to simulate the application of 180 N in models mimicking animals with different dimensions, muscle masses, and distribution of the common perineal nerve, without the need to sacrifice additional animals. Subsequently, in vivo testing of the force defined here will be essential to validate its effectiveness in a real preclinical scenario. This will require an adaptation of the non-serrated crushing clamp that was previously used in the rat and sheep models. The authors intend to modify the tool and its spring to achieve the application of a force of 180 N for 1 min to induce a 5 mm crushing lesion in sheep, following the protocol previously described. This way, it will be possible to confirm that in addition to the functional deficits already achieved by lower forces, histologically and stereologically, there will be an effective axonotmesis with crushing of all nerve fibers and disruption of myelin wraps and connective tissue coatings. Considering the previous observations and results and the statistical differences observed, the authors believe in the effectiveness of the conditions established here.

## 5. Conclusions and Further Directions

The present study demonstrates that the application of a load of 180 N to the common peroneal nerve of sheep is capable of significantly influencing biomechanical parameters such as ultimate tensile load, stiffness, strain, and stress, with an evident loss of resistance that should be associated with complete crushing of the nerve fibers and destruction of structural elements of the nerve trunk. It is expected that the application of this force to nerves in vivo will be able to induce an effective and complete axonotmesis, thus allowing the standardization of a crush injury protocol in the sheep, which could be used in combination with the functional and postmortem evaluation protocols developed by the research group for this animal model.

## Figures and Tables

**Table 1:** Values of the biomechanical parameters of each nerve subjected to different crushing forces. Values expressed as Mean + SEM.

Crushing Force (N)	Stress (MPa)	Strain (%)	Ultimate Tensile Strength (N)	Displacement at Rupture (mm)	Stiffness (N/mm)
0 N	//	//	109.59 ± 6.33	11.12 ± 1.30	9.95 ± 0.98
80 N	3.17 ± 0.17	40.67 ± 14.39	91.16 ± 9.19	8.78 ± 1.60	10.56 ± 1.00
180 N	7.55 ± 0.43	58.57 ± 14.30	49.38 ± 19.13	7.27 ± 1.39	6.26 ± 0.96

## Data Availability

The data that support the findings of this study are available from the corresponding author on request.
